# RETRACTION: CBX8 Interacts with Chromatin PTEN and Is Involved in Regulating Mitotic Progression

**DOI:** 10.1111/cpr.70083

**Published:** 2025-06-19

## Abstract

**RETRACTION**: ChoiB. H. , ColonT. M. , LeeE. , KouZ. , DaiW. , “CBX8 Interacts with Chromatin PTEN and Is Involved in Regulating Mitotic Progression,” Cell Proliferation 54, no. 11 (2021): e13110. 10.1111/cpr.13110.34592789 PMC8560621

The above article, published online on 15 December 2020 in Wiley Online Library (wileyonlinelibrary.com), has been retracted by agreement between the journal Deputy Editor, Yunfeng Lin; and John Wiley & Sons Ltd. A third party reported that the PTEN control bands in Figure 1A had previously been published in another article by some of the same authors (Choi et al. 2017 [https://doi.org/10.1186/s40164‐017‐0079‐0]). Author W. Dai responded to an inquiry by the publisher, but the authors were not able to locate the original data for further analysis. The publisher has confirmed that both articles report on different experimental conditions and time points for both sets of PTEN controls. The retraction has been agreed to because the duplication of the PTEN control data from an earlier publication fundamentally compromises the reliability of the reported results. The authors did not respond to our notice regarding the retraction.

**RETRACTION**: B. H. Choi , T. M. Colon , E. Lee , Z. Kou , W. Dai , “CBX8 Interacts with Chromatin PTEN and Is Involved in Regulating Mitotic Progression,” Cell Proliferation 54, no. 11 (2021): e13110. 10.1111/cpr.13110.34592789 PMC8560621

The above article, published online on 15 December 2020 in Wiley Online Library (wileyonlinelibrary.com), has been retracted by agreement between the journal Deputy Editor, Yunfeng Lin; and John Wiley & Sons Ltd. A third party reported that the PTEN control bands in Figure 1A had previously been published in another article by some of the same authors (Choi et al. 2017 [https://doi.org/10.1186/s40164‐017‐0079‐0]). Author W. Dai responded to an inquiry by the publisher, but the authors were not able to locate the original data for further analysis. The publisher has confirmed that both articles report on different experimental conditions and time points for both sets of PTEN controls. The retraction has been agreed to because the duplication of the PTEN control data from an earlier publication fundamentally compromises the reliability of the reported results. The authors did not respond to our notice regarding the retraction.

